# The alcohol harm paradox: using a national survey to explore how alcohol may disproportionately impact health in deprived individuals

**DOI:** 10.1186/s12889-016-2766-x

**Published:** 2016-02-18

**Authors:** Mark A. Bellis, Karen Hughes, James Nicholls, Nick Sheron, Ian Gilmore, Lisa Jones

**Affiliations:** 1 College of Health and Behavioural Sciences, Bangor University, Bangor, LL57 2PZ UK; 2 Public Health Wales, Hadyn Ellis Building, Cardiff University, Maindy Road, Cardiff, CF24 4HQ UK; 3 Centre for Public Health, Liverpool John Moores University, 15-21 Webster Street, Liverpool, L3 2ET UK; 4 Centre for History in Public Health, London School of Hygiene and Tropical Medicine, 15-17 Tavistock Place, London, WC1H 9SH UK; 5 Faculty of Medicine, University of Southampton, Mailpoint 811, University Hospital Southampton, Southampton, SO16 6YD UK; 6 School of Medicine, University of Liverpool, Cedar House, Ashton Street, Liverpool, L69 3GE UK

**Keywords:** Alcohol, Deprivation, Inequalities, Disease, Injury, Binge

## Abstract

**Background:**

Internationally, studies show that similar levels of alcohol consumption in deprived communities (vs. more affluent) result in higher levels of alcohol-related ill health. Hypotheses to explain this alcohol harm paradox include deprived drinkers: suffering greater combined health challenges (e.g. smoking, obesity) which exacerbate effects of alcohol harms; exhibiting more harmful consumption patterns (e.g. bingeing); having a history of more harmful consumption; and disproportionately under-reporting consumption. We use a bespoke national survey to assess each of these hypotheses.

**Methods:**

A national telephone survey designed to test this alcohol harm paradox was undertaken (May 2013 to April 2014) with English adults (*n* = 6015). Deprivation was assigned by area of residence. Questions examined factors including: current and historic drinking patterns; combined health challenges (smoking, diet, exercise and body mass); and under-reported consumption (enhanced questioning on atypical/special occasion drinking). For each factor, analyses examined differences between deprived and more affluent individuals controlled for total alcohol consumption.

**Results:**

Independent of total consumption, deprived drinkers were more likely to smoke, be overweight and report poor diet and exercise. Consequently, deprived increased risk drinkers (male >168–400 g, female >112–280 g alcohol/week) were >10 times more likely than non-deprived counterparts to drink in a behavioural syndrome combining smoking, excess weight and poor diet/exercise. Differences by deprivation were significant but less marked in higher risk drinkers (male >400 g, female >280 g alcohol/week). Current binge drinking was associated with deprivation independently of total consumption and a history of bingeing was also associated with deprivation in lower and increased risk drinkers.

**Conclusions:**

Deprived increased/higher drinkers are more likely than affluent counterparts to consume alcohol as part of a suite of health challenging behaviours including smoking, excess weight and poor diet/exercise. Together these can have multiplicative effects on risks of wholly (e.g. alcoholic liver disease) and partly (e.g. cancers) alcohol-related conditions. More binge drinking in deprived individuals will also increase risks of injury and heart disease despite total alcohol consumption not differing from affluent counterparts. Public health messages on how smoking, poor diet/exercise and bingeing escalate health risks associated with alcohol are needed, especially in deprived communities, as their absence will contribute to health inequalities.

## Background

Alcohol is responsible for 5.1 % of the global burden of disease and injury (disability adjusted life years) and 3.3 million deaths worldwide [1]. Such disease and death result from over 200 conditions either entirely or partially associated with alcohol consumption [1]. For most conditions, there is a positive ordinal relationship between consumption and increased risk [2, 3] with a few conditions (e.g. ischaemic stroke and heart disease) recording U- and J-shaped relationships [4–6]. While the latter suggest some health benefits from low alcohol consumption the validity and extent of such benefits remain contested [7]. The ordinal relationship between alcohol consumption and harm is also complicated by deprivation. A range of studies identify that deprived communities suffer substantively greater alcohol-related morbidity and mortality despite reporting average alcohol consumption similar to their more affluent counterparts [8–11]. While greater polarisation (i.e. more abstainers and more heavy drinkers) in deprived populations may account for some differences, deprived drinkers appear to suffer greater harms even after accounting for ecological confounders [1]. The mechanisms underpinning this alcohol harm paradox remain unclear. However, a range of different hypotheses can be postulated.

One plausible explanation is that deprived populations are exposed to other health challenges (e.g. though poorer diet and smoking) that interact especially with higher levels of alcohol consumption to create a multiplicative (i.e. synergistically harmful) increase in morbidity (combined health challenges hypotheses). Thus, obesity and higher levels of regular alcohol consumption interact to increase risks of liver disease mortality to a greater extent than the sum of each individual risk [12]. Equally, alcohol and smoking also show similar interactions associated with, for instance, increased risk of cancer (e.g. laryngeal [13]).

The next possible explanation postulates that while total alcohol consumption may be similar in deprived and more affluent communities there may be epidemiologically relevant differences in patterns of consumption (e.g. bingeing) and types of alcohol consumed (drinking pattern hypotheses). Thus, even occasional heavy drinking sessions (>60 g pure alcohol at least monthly) remove any benefits from reduced risks of ischaemic heart disease (IHD) that individuals might otherwise accrue from moderate drinking [14]. Consumption of the same amount of alcohol but in fewer sessions is also associated with increased risks of injury [3]. However, for liver disease the relative merits of bingeing rather than consuming the same amount of alcohol over more days are unclear [15, 16]. Further, types of alcohol consumed may also carry additional health risks with for instance spirit consumption in some populations having been associated with greater risks of cirrhosis, IHD and certain cancers [17, 18].

Drinking histories hypotheses propose that deprived individuals currently drinking similar quantities of alcohol have important differences in their historical drinking patterns. Thus, increased risks of alcohol-related cancers continue in individuals who have adopted abstinence from alcohol for over a decade (oesophageal, head and neck cancers [19]). The drinking history of deprived compared to more affluent individuals may differ both in terms of previously consuming more alcohol (including through starting drinking at an earlier age) or consuming greater proportions of total consumption though heavy or binge drinking sessions.

A final plausible hypothesis is that rather than the alcohol harm paradox being true, individuals in more deprived communities may actually drink more than their affluent counterparts but underestimate actual consumption through forgetting drinking occasions, poor recall of drinks per drinking session and underestimation of drink size [20–23]. In the UK only around 60 % of all alcohol sold for consumption is accounted for in national drinking surveys [24]. However, how underestimation of alcohol consumption varies with deprivation is poorly understood.

Here we use a national survey (*n* = 6015) of alcohol consumption enhanced to measure differences in the current and historic drinking patterns of poorer and more affluent drinkers who currently consume similar total amounts of alcohol. Using results from these analyses combined with epidemiological information from other studies we assess which factors may explain the alcohol harms paradox.

## Methods

Survey inclusion criteria were individuals aged 16 years or over and resident in England. A target sample size of 6000 was set and telephone interviews were conducted between May 2013 and April 2014. Sampling used a random probability method where English landline numbers were randomly selected (by a commercial company) from a national stratified database to allow equal representation across all English regions. Regionally stratified sampling was not possible for mobile phone numbers. Random Digit Dialing was then used to call phone numbers (see Bellis et al., 2015 [24] for full details). For all calls, respondents’ postcodes were recorded at interview and converted into lower super output areas (LSOAs; geographical areas with a population mean of 1500 [25]) of residence. Each respondent was assigned a measure of deprivation (Index of Multiple Deprivation 2010; IMD [26]) based on nationally published IMDs for each LSOA. IMD combines 38 separate indicators, grouped into seven domains (income, employment, health, education, crime, access to services and living environment) to create a single measure of deprivation. All data sets used in the compilation of the IMD have been subject to quality assurance and the methodology used to create the index independently reviewed [27]. IMD and other ecological measures of deprivation have previously been used to identify socio-demographic variations in alcohol harms across England [8]. For the purpose of examining differences between deprived and more affluent populations, IMDs were dichotomised into those in the poorest two quintiles (deprived) nationally and those in the other three (non-deprived; Table 1).

**Table 1:** Variations in typical alcohol consumption status with sample demographics

		All		Alcohol consumption categories^a^						
				Minimal consumer %	Lower risk %	Increased risk %	Higher risk %	Never drank %	Ex-drinker %	*P*
		*n*	6015	1275	2429	682	165	344	1120	
Age (years)	18–34	703	11.7	18.8	50.5	8.8	3.4	8.7	9.8	
	35–54	1711	28.5	18.7	43.8	13.4	2.8	5.7	15.6	
	55–74	2668	44.4	21.9	39.3	12.3	3.0	3.7	19.8	
	75+	933	15.5	25.5	29.6	6.9	1.4	9.3	27.3	<0.001
Sex	Female	3888	64.6	26.1	35.8	9.9	1.7	6.3	20.3	
	Male	2127	35.4	12.2	48.8	14.0	4.8	4.7	15.6	<0.001
Ethnicity^b^	White	5601	93.1	21.6	41.7	11.8	2.8	3.6	18.5	
	Asian/Chinese	188	3.1	10.1	14.4	1.1	1.1	55.3	18.1	
	Black/Other/not stated	226	3.8	19.9	28.8	9.7	2.2	16.4	23.0	<0.001
Deprived	No	4045	67.3	20.6	43.1	12.6	2.6	4.4	16.7	
	Yes	1970	32.8	22.4	34.8	8.8	3.1	8.4	22.5	<0.001

^a^Minimal, male & female >0–1 unit; lower risk, male >1–21 units, female >1–14 units; increased risk, male >21–50 units, female >14–35 units; higher risk, male >50 units, female >35 units. One UK unit is approximately 8 g of pure alcohol. ^b^White includes White British, Irish and other; Asian/Chinese includes Indian, Pakistani, Bangladeshi, Asian other or Asian British and Chinese; Black/other/not stated includes; Black or Black British, African, Caribbean, Black African Caribbean, other and not stated

Phone numbers were called up to seven times (Monday, Wednesday, Friday, 9.30 am to 5.30 pm; Tuesday, Thursday, 9.30 am to 9.00 pm; Saturday, 10.00 am to 4.00 pm Saturday). Any no answers, call back requests or answer machines were called until a respondent either agreed or declined to participate or the study end date was reached. A total of 97,805 calls were made of which 71,621 resulted in a discontinued phone line, a contact (e.g. business premises) that was not within the sampling frame or a no answer and subsequent follow up of up to seven repeat calls to the same number. Of those individuals contacted 6092 agreed and 20,092 refused to participate in the study (i.e. a response rate of 23.3 %). For this study, data were limited to 6015 individuals aged 18 years or above who provided full demographic and current alcohol consumption data.

Respondents were asked their age, sex and ethnicity (according to Office of National Statistics categories [28]). Due to small numbers in some ethnicities, ethnic categories were reduced to White, Asian/Chinese, and Black/other/preferred not to say (see Table 1 for more details). Individuals were classified as current alcohol consumers (drank in the last 12 months) or abstainers (grouped into those who had never drank and those who had quit). For current drinkers, typical alcohol consumption was determined using a question on typical frequency of alcohol consumption combined with detailed questions on types, locations and quantities of alcohol consumed on typical drinking days [24]. Consumers were categorised according to weekly drinking levels consistent with those used by national statistics (UK units, where 1 unit is approximately 8 g of pure alcohol; categories – minimal, male and female, >0–1 unit; lower risk, male >1–21, female >1–14 units; increased risk, male >21–50, female >14–35 units; higher risk, male >50, female >35 units [29]). Hypotheses were tested in three groupings: combined health challenges (interactions between alcohol consumption and other health challenging behaviours); current drinking patterns (measures of bingeing, types of alcohol consumed and unreported alcohol consumption) and drinking histories (age of initiation of drinking and drunkenness and history of frequent and drunkenness/binge drinking).

Combined health challenges were explored using dichotomised variables: current smoker (daily or occasional); poor diet (averaging ≤1 portion of fruit or vegetables per day); typically low exercise (<1 exercise session long enough to work up a sweat or get out of breath per week); overweight (self-reported height and weight equating to Body Mass Index >25). Current drinking pattern hypotheses were tested by examining the types of alcohol consumed on typical drinking occasions (categories: wine, beer/cider, spirits) with individuals being able to select more than one type. A derived variable was created as a proxy for typically binge drinking (versus distributed drinking patterns). Thus, total annual consumption was divided by drinking frequency and males averaging >8 units and females >6 units per drinking session were classified as binge drinkers [30]. A final variable used for assessing drinking pattern measured missing or typically unreported alcohol consumption. Thus, the survey tool also collected information on atypical or special occasion drinking not usually collected in typical drinking surveys [24]. Individuals were divided into those whose atypical/special occasion drinking did and did not add ≥5 units (i.e. ≥40 g of pure alcohol) to their weekly consumption.

Drinking history hypotheses were tested by questions addressing age at which individuals first started to drink regularly (defined as at least once or twice a month) and age at which they first drank enough to feel drunk (defined as slurred speech or unsteady on feet). Both variables were dichotomised to identify individuals reporting the drinking behaviour before 18 years of age. Finally, two sets of questions asked individuals their frequency of drinking and frequency of drunkenness/bingeing (defined as 5+ drinks in a session) when aged 18 and 30 years. Here, for individuals aged ≥35 years only, variables were dichotomised to identify those who reported drinking most days of the week (four or more) when aged both 18 and 30 years and also into those who reported being drunk/bingeing at least monthly when aged both 18 and 30 years.

The survey was piloted on 840 individuals between November 2012 and February 2013 and minor changes were made to the wording of questions and prompts provided by surveyors in order to improve clarity for respondents. Responses were recorded in a computer-assisted telephone interview system with data then transferred to SPSS v21 for analysis. Chi-squared analyses are used to examine relationships between alcohol harm paradox variables (i.e. combined health challenges, drinking patterns and drinking history variables) and deprivation within groups consuming similar quantities of alcohol. T tests are used to compare means and binary logistic regression is employed to control for demographic confounders and additionally explore how each alcohol harm paradox variable is associated with interactions between deprivation and alcohol consumption.

Ethical approval for the study was obtained from Liverpool John Moores University’s Research Ethics Committee. The voluntary and anonymous nature of the study was explained to all participants as part of obtaining informed consent.

## Results

Table 1 provides the overall demographics and typical drinking categories of survey participants. Typical drinking differed between deprived and non-deprived respondents, with lower and increased risk drinking more common in non-deprived individuals and minimal, never and ex-drinkers associated with deprivation. Significant differences were also apparent by age, sex and ethnicity with, for example, males typically drinking more heavily than females (Table 1). Within alcohol consumption categories, there were no significant differences in mean units consumed per week by deprivation.

Table 2 examines differences in alcohol harm paradox hypothesis variables between deprived and non-deprived individuals who consume similar amounts of alcohol. In all consumption categories except never drank, individuals in the deprived group are more likely to smoke. The difference is most marked in increased risk and higher risk consumption categories where smoking prevalence in both is 2.4 times higher in deprived individuals. A similar pattern is also shown with fruit and vegetable consumption. Differences in exercise between deprived and non-deprived individuals by alcohol consumption category were less marked; although low levels of exercise were still more frequent in deprived minimal and higher risk drinkers (versus non-deprived drinkers in the same categories). Deprived individuals were more likely to be overweight in all consumption categories except higher risk and never drinkers.

**Table 2:** Associations between deprivation and alcohol harm paradox variables stratified by alcohol consumption

Alcohol harm paradox variables	Deprived	*n*	All	Alcohol consumption category					
				Minimal consumer	Lower risk	Increased risk	Higher risk	Never drank	Ex-drinker
Combined health challenges									
Current smoker %	No	4041	12.1	12.1	10.1	12.4	19.1	9.6	16.4
	Yes	1966	21.0	18.8	19.0	29.9	45.8	10.4	23.5
	*P*		<0.001	0.001	<0.001	<0.001	<0.001	0.801	0.003
< =1 portion fruit or veg/day %	No	4037	8.5	8.8	6.5	5.0	17.1	13.5	13.2
	Yes	1967	16.2	12.7	12.7	14.4	40.7	21.2	20.5
	*P*		<0.001	0.032	<0.001	<0.001	<0.001	0.064	0.001
< 1 exercise session/week %	No	4037	27.4	31.3	21.8	19.1	23.8	39.3	40.6
	Yes	1967	33.5	37.6	25.1	23.6	39.0	33.5	45.7
	*P*		<0.001	0.023	0.087	0.210	0.040	0.312	0.091
Overweight BMI >25 %	No	3757	51.5	52.8	48.4	51.9	60.2	48.3	57.0
	Yes	1806	60.2	65.0	56.3	63.4	58.3	51.8	63.3
	*P*		<0.001	<0.001	<0.001	0.011	0.816	0.560	0.044
Drinking patterns^a^									
Average session binge %	No	3640	17.9	1.0	16.5	36.0	87.6		
	Yes	911	25.0	2.0	27.1	51.7	91.7		
	*P*		<0.001	0.111	<0.001	<0.001	0.422		
Typically drink wine %	No	3189	64.0	54.9	65.2	74.8	63.8		
	Yes	1362	47.9	47.1	48.4	54.6	30.0		
	*P*		<0.001	0.008	<0.001	<0.001	<0.001		
Typically drink spirits %	No	3189	16.4	16.7	15.7	16.5	24.8		
	Yes	1362	22.6	22.2	22.3	21.3	33.3		
	*P*		<0.001	0.017	<0.001	0.168	0.280		
Typically drink beer/cider %	No	3189	26.5	18.3	28.0	30.3	47.6		
	Yes	1362	34.6	21.7	37.3	48.9	56.7		
	*P*		<0.001	0.136	<0.001	<0.001	0.263		
> =5 units/week underestimate %	No	3189	16.0	3.4	16.8	31.7	28.6		
	Yes	1362	13.5	2.0	16.3	25.9	30.0		
	*P*		0.031	0.180	0.800	0.153	0.860		
Drinking histories^b^									
Regular drinker <18 years %	No	3501	41.8	40.4	41.4	47.0	58.1		36.4
	Yes	1581	43.4	37.8	46.4	48.0	61.0		37.6
	*P*		0.284	0.410	0.025	0.830	0.715		0.721
Drunk <18 years %	No	3196	43.2	35.4	45.2	51.0	57.1		35.8
	Yes	1470	45.1	38.4	49.8	51.8	60.0		35.9
	*P*		0.226	0.352	0.053	0.852	0.728		0.975
Frequent drinking history %	No	3381	4.8	1.2	5.2	9.4	6.6		4.3
	Yes	1511	4.9	1.3	5.2	15.3	9.1		3.5
	*P*		0.838	0.889	0.998	0.049	0.728		0.522
Drunken/bingeing history %	No	3397	13.3	7.3	14.6	20.7	31.5		9.8
	Yes	1511	15.4	6.3	19.1	35.1	40.0		8.9
	*P*		0.052	0.549	0.013	<0.001	0.325		0.650

^a^Analysis possible only for current drinkers. ^b^Analysis possible only for ex and current drinkers; For definitions of each alcohol harm paradox variable please see methodology. Consumption categories are defined as in Table 1. Frequent drinking and drunken/bingeing histories are limited to individuals aged 35 years or more at interview. One unit is approximately 8 g of pure alcohol

Deprived lower and increased risk drinkers were more likely to be binge drinkers than their non-deprived counterparts (Table 2). Further, deprived drinkers were less likely to typically consume wine across all drinker categories. Overall, deprived individuals were more likely to typically consume spirits and beer/cider (Tables 2 and 3). However, disproportionate increases were only significant for beer/cider in increased risk drinkers (Table 3). There were no significant differences by deprivation in under-reporting of alcohol consumption in any individual drinking category (Table 2). Overall however, under-reporting was marginally higher in non-deprived individuals (Table 3). Finally, for drinking histories, deprivation was associated with regularly drinking under 18 years of age in lower risk drinkers only. No consumption category showed differences in age when first drunk by deprivation. Deprivation was only marginally associated with having a history of frequent drinking in increased risk drinkers (Table 2). However, a history of drunkenness/bingeing was higher in deprived lower and especially increased risk drinkers compared to their non-deprived counterparts.

**Table 3:** Logistic regression: relationships between alcohol paradox variables and deprivation alone and interacting with alcohol consumption

Alcohol harm paradox variables	Deprivation (ref non-deprived)			Deprived v. non-deprived within alcohol consumption category (ref minimal consumer^a^)															
	Deprived				Lower risk			Increased risk			Higher risk			Never			Ex-drinker		
	AOR +/−95%CI		*P*	*P* ^b^	AOR +/−95%CI		*P*	AOR +/−95%CI		*P*	AOR +/−95%CI		*P*	AOR +/−95%CI		*P*	AOR +/−95%CI		*P*
Combined health challenges																			
Current smoker	1.86	1.52–2.28	***	*	1.23	0.82–1.85	ns	1.86	1.09–3.17	*	2.23	1.01–4.89	*	0.57	0.26–1.25	ns	0.94	0.61–1.47	ns
< =1 portion fruit-veg/day	1.87	1.58–2.22	***	ns	1.37	0.85–2.20	ns	1.99	0.99–3.98	ns	2.12	0.93–4.85	ns	0.95	0.48–1.90	ns	1.07	0.65–1.76	ns
< 1 exercise session/week	1.38	1.22–1.56	***	ns	0.89	0.64–1.24	ns	0.93	0.57–1.53	ns	1.49	0.70–3.16	ns	0.60	0.35–1.03	ns	0.90	0.63–1.29	ns
Overweight BMI >25	1.44	1.28–1.63	***	ns	0.88	0.64–1.20	ns	0.96	0.61–1.51	ns	0.60	0.29–1.22	ns	0.86	0.50–1.47	ns	0.80	0.55–1.14	ns
Drinking patterns																			
Average session binge	1.73	1.44–2.07	***	ns	0.88	0.33–2.36	ns	0.97	0.35–2.73	ns	0.8	0.18–3.48	ns						
Typically drink wine	0.42	0.34–0.53	***	**	0.65	0.48–0.89	**	0.55	0.34–0.86	*	0.30	0.14–0.65	**						
Typically drink spirits	1.43	1.21–1.68	***	ns	1.04	0.72–1.50	ns	0.96	0.57–1.63	ns	1.00	0.46–2.14	ns						
Typically drink beer/cider	1.57	1.34–1.85	***	ns	1.36	0.92–2.00	ns	1.99	1.18–3.36	*	1.16	0.52–2.61	ns						
> =5units/week underestimate	0.79	0.65–0.96	*	ns	1.58	0.71–3.52	ns	1.27	0.54–3.00	ns	1.85	0.65–5.26	ns						
Drinking histories																			
Regular drinker <18 years	0.99	0.83–1.18	ns	ns	1.35	0.97–1.87	ns	1.15	0.73–1.80	ns	1.27	0.62–2.63	ns				1.17	0.78–1.75	ns
Drunk <18 years	1.00	0.82–1.20	ns	ns	1.01	0.71–1.45	ns	0.90	0.56–1.45	ns	0.92	0.43–1.98	ns				0.83	0.53–1.30	ns
Frequent drinking history^c^	1.09	0.73–1.63	ns	ns	0.96	0.29–3.19	ns	1.59	0.46–5.53	ns	1.35	0.24–7.68	ns				0.76	0.21–2.78	ns
Drunken/bingeing history^c^	1.20	0.95–1.52	ns	*	1.87	1.06–3.31	*	2.60	1.32–5.14	**	1.59	0.64–3.93	ns				1.11	0.56–2.23	ns

Age, sex, alcohol consumption risk category (alone) and ethnicity were all also included in the logistic regression model. However, for reasons of space only the key variables deprivation and the deprivation interaction with alcohol consumption risk category are shown. ^a^Minimal is the reference category for interactions between deprivation and alcohol consumption. ^b^ *P* values shown refer to the significance of the overall contribution of the interactive term (Alcohol Consumption Risk Category*Deprivation) to the model. ^c^Frequent drinking and drunken/bingeing histories are limited to individuals aged 35 years or more at interview (See Methods for more details). For deprivation alone AORs (Adjusted Odds Ratios) use non-deprived as the reference category. 95 % CI = 95 % Confidence Intervals. *BMI* Body Mass Index. Consumption categories are defined as in Table 1. 1 unit is approximately 8 g of pure alcohol. *p<0.05, **p<0.01, ***p<0.001.

Table 3 provides results from logistic regression analyses. Results are presented for the independent relationship between each alcohol harm paradox variable and deprivation alone as well as for the interaction between deprivation and current drinking status. The latter is included to examine if the distribution of, for instance, current smoking differs significantly between deprived and non-deprived groups with changes in drinking status category. For combined health challenges, current smoking, poor diet, low exercise and being overweight are all strongly linked with deprivation (Table 3). Taking the interaction between deprivation and current drinking behaviours into account, however, current smoking is disproportionately elevated in deprived drinkers who are increased and higher risk drinkers (compared with elevations in minimal drinkers). Although similar associations were apparent for diet they just failed to reach significance (Table 3).

For current drinking patterns, typically consuming spirits and beer/cider and not typically consuming wine are also associated with deprivation (Table 3). The negative relationship between typically consuming wine and deprivation is proportionally greater across all consumption categories (compared with minimal drinkers). While overall more deprived individuals typically consumed spirits, this effect did not vary with consumption category (Table 3). The overall effect of more typical consumption of beer/cider in deprived individuals was significantly elevated in increased risk alcohol consumers (Table 3). Higher identification of underreporting alcohol consumption (≥5 units/week) was marginally associated with more affluence overall but with no variation by drinking category. Bingeing was associated overall with deprivation but this relationship was also not modified by its interaction with any consumption category (Table 3). Finally, for drinking history variables there were no direct associations with overall deprivation. However, a history of drunkenness /bingeing (based on point estimates at 18 and 30 years of age) was associated with deprivation in lower and increased risk alcohol consumers (Table 3).

**Fig. 1:**
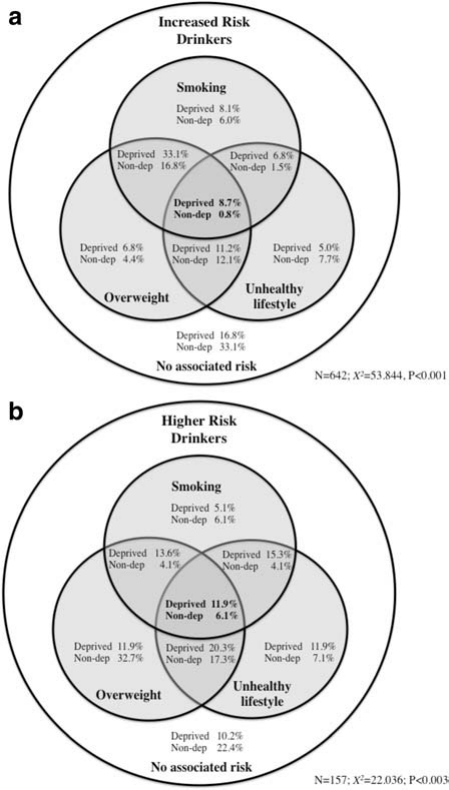
Venn diagram of overlap between smoking, unhealthy lifestyle and being overweight in (**a**) increased risk drinkers and (**b**) higher risk drinkers, stratified by deprivation. Footnote: Unhealthy lifestyle is calculated as having low levels of either exercise or poor diet (see Results for more details). Chi squared statistics compare the distribution of deprived and non-deprived (Non-dep) drinkers across the Venn diagram categories

With a substantial number of combined health challenges variables linked to deprivation the potential cumulative impact of these is further explored. Using a derived variable *unhealthy lifestyle* (calculated as having either low levels of exercise or poor diet) along with the variables current smoking and excess weight, the cumulative health challenges in deprived and non-deprived increased risk drinkers (Fig. 1a) and higher risk drinkers (Fig. 1b) were compared. Among increased risk drinkers, two thirds (66.9 %) of non-deprived respondents have at least one other health challenge (current smoking, excess weight, unhealthy lifestyle), compared with 83.2 % of those living in deprived areas. Less than 1 % of non-deprived increased risk drinkers have all three health challenges increasing to nearly 9 % of their deprived counterparts. Further, among those increased risk drinkers with at least one other health challenge (Fig. 1a, greyed area), 37.3 % in the non-deprived group binge drink compared with 56.7 % in the deprived group (*X* ^2^ = 14.605, *P* < 0.001). Differences in the distribution of combined health challenges (between deprived and non-deprived groups) were similar but less marked in higher risk drinkers. However, differences in binge drinking levels (by deprivation) amongst those with least one other health challenge (Fig. 1b, greyed area) were not significant (94.34 vs. 90.69 %, *X* ^2^ = 0.550, *P* = 0.458).

## Discussion

Both in the UK and internationally, similar alcohol consumption levels have been associated with greater impacts on the health of more deprived individuals. In a systematic review of socio-economic differences in alcohol-attributable mortality Probst et al. suggest that the poorer diet of individuals living in deprivation (e.g. more high fat and salt foods and less fruit and vegetable consumption) may interact with alcohol consumption to alter protein and vitamin absorption and increase risks of health harms [31]. They also acknowledge that interactions between higher smoking prevalence in deprived areas and alcohol consumption may contribute to an increased risk of some cancers. Moreover, they suggest that poorer access to primary care may contribute to greater harms from alcohol in deprived groups. Consequently, individuals with lower socio-economic status may face cost, transport, availability and stigma-related issues that restrict their access to services which might help with alcohol- related problems [31–33]. Along with an increasing literature describing the disproportionate impact of alcohol on more deprived communities advocacy to address such health inequalities is also developing at national and international levels [34]. Generally, however, alcohol consumption surveys typically do not collect sufficient current and historic data to test competing explanations for this alcohol harm paradox. Consequently, using a bespoke national survey we have examined how combined health challenges, current drinking patterns and historical drinking behaviours differed with deprivation between individuals who currently consume similar quantities of alcohol.

Results confirm strong associations between drinking and smoking (Table 2) and specifically identify a disproportionate concentration of smokers in increased and higher risk alcohol consumers from deprived communities (Table 3). In addition such deprived individuals are more likely to be overweight and have unhealthier lifestyles. Consideration of alcohol-related health harms often focuses on higher risk drinkers and results here suggest such individuals (males >50 units or 400 g of pure alcohol/week; females >35 units or 280 g of pure alcohol/week) in deprived communities face combined health challenges likely to have a multiplicative impact on health. Such impacts include increased risks of conditions specifically associated with alcohol (e.g. alcohol-related liver disease) and those where alcohol is one of many multifactorial causes (e.g. oesophageal cancer, breast cancer, hypertension and macular degeneration) [3, 35–37]. Arguably, less attention is paid to increased risk drinkers (here males >21–50 units or >168–400 g of pure alcohol/week; females >15–35 units, >112–280 g of pure alcohol/week). However, across England 18 % of men and 13 % of women report drinking at increased risk levels (vs. 5 and 3 % at higher risk levels respectively) [29] and the contribution of increased and even lower risk drinkers to overall harms is substantive. For instance only around a fifth of alcohol-related breast cancer deaths are in women drinking ≥35 units (≥280 g of pure alcohol) a day with the rest in those drinking at lower consumption levels (England [38]). For many conditions multiplicative impacts are still relatively poorly defined. However, the combined risk from smoking with alcohol consumption may be more than double that expected from the summed risks from smoking and alcohol in the absence of synergies [39]. In this study increased risk drinkers in deprived communities were 10.9 times more likely to carry the additional burden of not just smoking but also unhealthy lifestyle and excess weight (Fig. 1).

As well as combined health challenges, results suggest that individuals in deprived groups may differ from those in non-deprived groups (with similar current total weekly alcohol consumption) in their choice of alcohol types and both current binge and historical binge drinking (Tables 2 and 3). Deprived drinkers are less likely to typically consume wine and more likely to consume beer or spirits (Table 3). Following extensive debate over the Mediterranean diet and relative benefits of wine consumption compared with other alcoholic drinks [40], more recent epidemiological analyses appear to offer some support for health challenges differing by drink type [41–44]. Further, we found individuals from deprived groups also appear to consume alcohol in fewer but heavier drinking sessions (Tables 2 and 3). Moreover, based on retrospective estimates of frequency of heavy drinking (at ages 18 and 30 years) deprived individuals are also more likely to have previously been drunk/bingeing, although differences from more affluent individuals are limited mainly to lower and increased risk drinkers (Tables 2 and 3). Consuming similar amounts of alcohol in fewer sessions increases risks of alcohol-related injuries (including unintentional and violent [35, 45]) and critically can also eradicate any potential protection moderate drinking might offer from IHD. Consequently, higher bingeing in deprived groups (both historically and currently) is consistent with these populations suffering more injury and IHD than more affluent drinkers despite current total alcohol consumption being the same. The long-term impact of a history of more frequent binge drinking is still poorly understood but any impact on life-time risks of IHD, cancers or other alcohol-related conditions is likely to be exacerbated by ex-drinkers (but not never drinkers) in deprived communities continuing to carry higher combined health challenges (smoking, poor diet and excess weight; Table 2) than ex-drinkers from more affluent groups.

There are a number of important limitations to this study. Response rate was 23.3 % and we cannot quantify any bias introduced by differences between individuals who agreed or declined to participate. We could not distinguish unoccupied properties from those where individuals chose not to answer their phones. Individuals who chose not to answer calls also represent a potential source of bias in the final sample which we cannot quantity. Typical response rates for telephone surveys are declining with one major US provider tracking falls from 28 % compliance in 2000 to rates well below those achieved here (i.e. around 9 %) in 2012 [46] Consequently, while response related bias remains a potential confounder, compliance here is well within the range experienced elsewhere. Inevitably alcohol harm paradox variables were proxy measures. Thus, our history of drunkenness/bingeing and of frequent drinking was limited to retrospective measures for ages 18 and 30 years. We cannot establish how well they correlate with all consumption over this 12 year period or any other period in respondents’ drinking histories and how any recall error may have impacted results. Accuracy of recall is also a potential issue for age at which regular drinking began and age when first drunk. Our measure of deprivation was ecological and relied on assigning individuals an average level of deprivation according to their area of residence. This methodology has the benefit of using a composite of multiple quality-assured measures of deprivation. However, ecological categorisation inevitably means individuals with different personal deprivation characteristics can be classified within the same category. Such classification may have hidden significant relationships between deprivation and some of the main variables of interest. Consideration of questionnaire length and compliance precluded us incorporating a comprehensive set of deprivation measures in this survey. However, future studies of the alcohol harm paradox would benefit from examining both individual and ecological measures. We also used a measure of atypical/special occasion drinking to identify unreported alcohol consumption. However, deprived and non-deprived individuals may have differed in recall of these occasions or assessed sizes and strengths of drinks differently [22]. Finally, we could not assess some alternative hypotheses for the alcohol harm paradox. Other competing theories include: individuals who become ill as a result of alcohol being drawn into more deprived communities through long-term disability and unemployment; genetic predisposition to suffering harms from alcohol in deprived populations; lower survey completion rates amongst heavy drinkers in poorer areas; and poorer access to and use of health and social support systems in deprived communities resulting in later or less treatment and support to avoid or tackle alcohol-related ill health [1, 9, 10].

## Conclusions

Continued alcohol consumption creates long-term stresses on the body’s immunological, neurological and hormonal systems. For alcohol consumers, individuals’ ability to achieve and maintain good health is inevitably compromised by other challenges such as excess weight, smoking and poor diet and exercise regimes. Extreme peaks in alcohol consumption appear to further reduce individuals’ resistance to IHD and increase short-term harms from injury. This study has identified both combined health challenges and current and historic binge drinking as factors associated with deprivation even when considering individuals who currently consume similar amounts of alcohol. While causality could not be identified such findings suggest at least that harms from alcohol consumption should not be viewed in isolation but seen as associated with a public health pattern or syndrome of health challenging behaviours disproportionately impacting deprived communities. Consequently, findings here combined with those reported elsewhere [31, 36] indicate national guidance on safer drinker levels should routinely inform individuals that those who smoke, are overweight or live unhealthy lifestyles may suffer greater harms from similar levels of alcohol consumption. Given such drinkers are more likely to reside in deprived communities the current absence of such information may contribute to health inequalities [8]. On a global basis alcohol industries are increasingly targeting developing countries for growth in alcohol markets [47]. Such countries often have high tobacco use [48], high levels of binge drinking [1] and generally poorer levels of health combined sometimes with high endemic levels of violence and injury [49] and low levels of health and social service support. To date consideration of the alcohol harm paradox has focused primarily on the disproportionate impacts of alcohol consumption on deprived individuals within more affluent countries. However, a better understanding of its broader implications to low and middle income countries is urgently needed as the health costs of increasing global alcohol consumption may be considerably higher than estimates from more affluent populations suggest.

## Abbreviations

Gms: grams
IHD: ischaemic heart disease
IMD: index of multiple deprivation
LSOA: lower super output area
